# The Augment of the Stability in Locking Compression Plate with Intramedullary Fibular Allograft for Proximal Humerus Fractures in Elderly People

**DOI:** 10.1155/2018/3130625

**Published:** 2018-09-16

**Authors:** Hua Chen, Peng Yin, Song Wang, Jiantao Li, Lihai Zhang, Kamran Khan, Licheng Zhang, Peifu Tang

**Affiliations:** ^1^Department of Orthopedics, Chinese PLA General Hospital, No. 28 Fuxin Road, Beijing 100853, China; ^2^Department of Orthopedics, Beijing Chaoyang Hospital, Capital Medical University, No. 8 GongTiNanLu, Beijing 100020, China; ^3^Medical College, Nankai University, No. 94 Weijin Road, Tianjin 300071, China; ^4^Department of Orthopedic Surgery, Massachusetts General Hospital and Harvard Medical School, Boston, MA, USA

## Abstract

**Objective:**

The objective of this study was to compare the clinical outcomes and complications between the locking compression plate (LCP) and LCP with fibular allograft in the treatment of patients with displaced proximal humerus fracture (PHF) in elderly people.

**Material and Methods:**

Between January 2010 and December 2013, a total of 97 elderly patients with displaced PHF were treated by LCP or LCP with fibular allograft, and finally 89 patients were included in our study. All the patients were divided into Group I (patients treated by LCP) and Group II (patients treated by LCP with fibular allograft). Function results were assessed by the disability of the arm, shoulder, and hand (DASH) score and Constant-Murley score (CMS), and complications were also recorded in each group.

**Results:**

The average follow-up was 35.2 months (range, 24-48 months) in Group I and 33.5 months (range, 24-48 months) in Group II. DASH in patients of Group I was significantly higher than that in patients in Group II and patients of Group I had CMS scores significantly lower than patients in Group II (P<0.05). The rates of varus malunion, screw perforation, and loss of reduction>5mm were significantly higher in Group I than in Group II (P<0.05).

**Conclusions:**

The present results showed that that patients treated by LCP with fibular allograft had a better functional outcome and a lower complication rate compared to patients treated by LCP alone. Suitable void filler in the proximal humerus for supporting the head fragment, medial cortical bone, and greater tuberosity might play a key role in reducing the incidence of the complications in elderly patients, especially with osteoporosis.

## 1. Introduction

Fractures of the proximal humerus account for 4%-5% of the whole body bone fractures [1, 2], and proximal humerus fracture (PHF) is the third common injury among the older people [3]. It has been reported that the incidence of PHF is increasing, especially in the older people [4, 5]. The majority of patients with stable or minimally displaced PHF could be treated conservatively [6, 7], but displaced PHF may require a surgical treatment in order to achieve fracture stability and allow for early motion [8]. Operative management of PHF still remains challenging for orthopedic surgeons in the world [8–11].

Various surgical techniques have been described for the unstable PHF, including tension banding [12, 13], intramedullary nailing [14–16], non-locking and locking plating [17–19], and shoulder hemiarthroplasty (HA) [20, 21]. Until now, there is still different consensus about the best treatment for PHF [22, 23]. Biomechanical data and clinical outcomes demonstrated that locking plating for displaced PHF is a promising treatment compared to other methods [24–28]. However, high complication rates of up to 49% in PHF patients by using locking plating method have been reported, and the most two common complications are varus malunion and screw perforation [9, 10, 29]. Therefore, many efforts have been made in order to overcome these problems, such as medial support screws [11, 30], cement augmentation [23, 31], additional medial plate [32], and bone autograft [33]. These treatments have partly decreased the complication rates, but meanwhile caused other problems, including humeral head necrosis [11], cement-related heat injuries [34], neurovascular injuries [32], and donor-site morbidity [35, 36].

Elderly people usually are accompanied by osteoporosis, and osteoporosis increases the difficulty of treating displaced PHF due to low bone mineral density (BMD) [37]. One study showed that the osteoporotic PHF was similar to the breakage of eggshell, and the contents in humeral head were nearly empty [38]. Therefore, effective augment of bone-to-implant construct is very critical. Biomechanical data demonstrated that a fibular allograft could increase the maximal failure loads of LCP fixation system and decrease the rate of varus collapse [37]. Additionally, fibular allograft could provide adequate bone stock, obtain easily, and avoid the donor-site morbidity [39]. In our pervious study [40], we adopted the technique of locking compression plate (LCP) with fibular allograft for the treatment of elderly four-part PHF, and the clinical results were favorable.

Although there are four biomechanical comparison studies on LCP and LCP with fibular allograft in PHF models [41–44], to our best knowledge, no comparative clinical study between LCP and LCP with fibular allograft for the treatment of PHF has been performed. Therefore, the aim of the present study was to compare the outcomes and complications of LCP and LCP with fibular allograft in elderly displaced PHF.

## 2. Material and Methods

Between January 2010 and December 2013, 97 patients with displaced PHF were operated by LCP or LCP with fibular allograft at our hospital. The inclusion criteria were (1) patients of age of 60 years or more, (2) acute unilateral closed three-part or four-part PHF, and (3) fracture fragments displaced more than 1 cm or angulated more than 45°. The exclusion criteria were (1) a history of shoulder surgery or chronic nonunion, (2) pathological or open fractures, and (3) complications of serious nervous or vascular injury. Finally, 89 patients were recruited in our study. The study was approved by our Institutional Review Board and written consent was obtained from all the subjects prior to participation in this study.

Appropriate clinical and radiological assessments were carried out for every patient before operation, and all patients received 1.5g cefuroxime preoperatively. All fractures were classified according to the Neer classification system [45]. All patients were then divided into two groups in line with different surgical techniques, and the patients operated by LCP alone were regarded as Group I, and the patients operated by LCP with fibular allograft were viewed as Group II.

In Group I, surgeries were performed through a standard deltopectoral approach [46], and a LCP ((Synthes, Switzerland) was placed in the fracture site after surgical reduction. At least 5 screws were fixed in the humeral head and the place of the screw tip was confirmed by an image intensifier during the operation. After meticulous irrigation, the incision was closed with a negative suction drain. In Group II, all the procedures were performed as our previous study [40]. In brief, operations were conducted through a standard deltopectoral approach, and the reduction of the humeral head and shaft was completed by laminar spreader under fluoroscopy. And then the fibular allograft, including fibular shaft or anatomical allograft, was inserted into the intramedullary canal from the lateral window of tuberosity fracture sites. In order to prevent the humeral head from varus displacement and deformation, the fibular allograft was pushed onto the medial calcar. After that, the greater tuberosity fragment was reduced and fixed, and then a LCP (Synthes, Switzerland) was fixed in the fracture sites. The locations of plate and screws were confirmed by fluoroscopy. If the fixation was satisfied, careful irrigation would be performed, and finally the incision was closed in layers with a negative suction drain.

**Figure 1:**
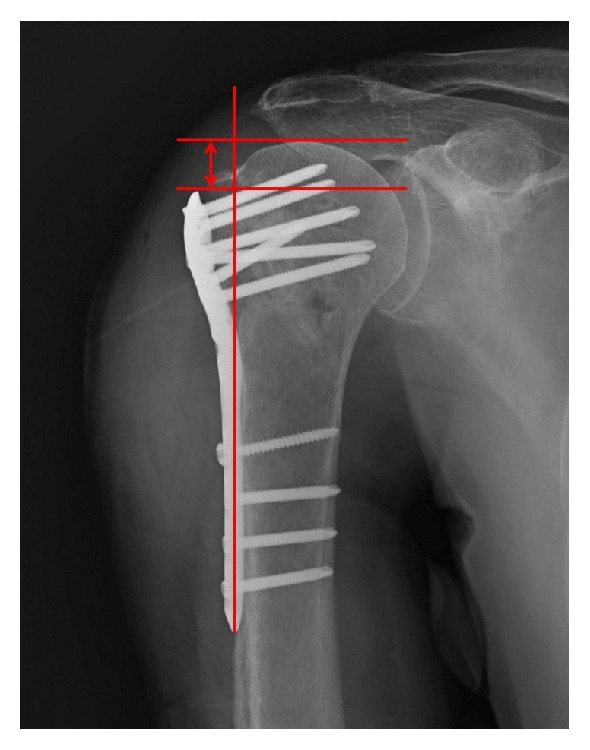
The illustration of the measurement of the height between the superior edge of the humeral head and the top edge of the proximal plate.

**Figure 2:**
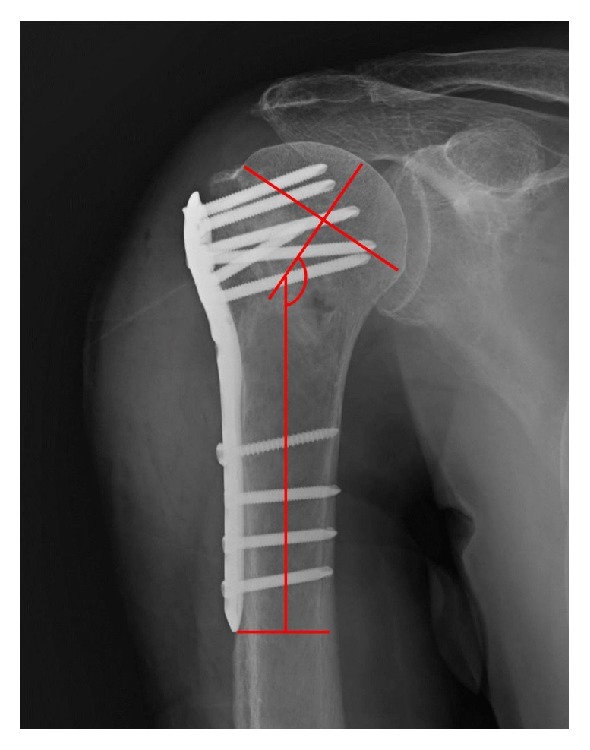
The illustration of the measurement of the humeral neck-shaft angle.

The “humeral head height” between the superior edge of the humeral head and the top edge of the proximal plate was measured on true anteroposterior (AP) radiographs of the shoulder, postoperatively and at 3-month follow-up, as Gardner's previous description [30] (Figure 1). A decrease of the height was interpreted as a loss of reduction. The humeral neck-shaft angle was measured as Agudelo's description [8] (Figure 2). In brief, a line was drawn from the superior to the inferior border of the articular surface, and then another line perpendicular to the previous line was drawn through the center of the humeral head. The angle between the perpendicular line and the line bisecting the humeral shaft was described as the humeral neck-shaft angle. After operation, restoration of the humeral neck-shaft angle up to 120°-150° was defined as anatomical reduction, acceptable reduction was defined as being between 110°and 120°, and malreduction was defined as being >150°or <110°[47].

Patients were immobilized in a sling postoperatively, and they begin to perform passive mobilization and pendulum exercises immediately. In addition, physiotherapy was carried out to all patients and gradually ceased around 3 weeks. The outcomes of operation based on patients' subjective rating were classified into four types as follows: excellent, good, fair, and poor. Function results were assessed by the disability of the arm, shoulder, and hand (DASH) score and Constant-Murley score (CMS). We employed dual-emission X-ray absorptiometry (DXA) to evaluate bone mineral density (BMD) for every patient. Radiographs, including true AP, axillary, and scapular Y views, were reviewed postoperatively and at 1 month, 2 months, 3 months, 6 months, 12 months, 24 months, and 48 months following operation. Complications, such as varus malunion, screw perforation, infection, and humeral head necrosis, were recorded during the follow-up. The data were analyzed by SPSS 15.0 software with independent* t*-test in continuous variables and chi-square and Fisher's exact test in nominal data. A statistically significant difference was determined when p<0.05.

## 3. Results

42 patients with LCP and 47 patients with LCP and fibular allograft were included in Group I and Group II, respectively. In Group I, there were 15 males and 27 females, with an average of 69.12 years (range, 60-85 years). All the injured arms were dominant. 18 patients suffered from medial comminution. The mean angle of the postoperative humeral neck-shaft was 128.71 degrees. According to the postoperative humeral neck-shaft angle, there were 34 patients with anatomical reduction, 7 patients with acceptable reduction, and 1 patient with malreduciton. In Group II, there were 12 males and 35 females, with an average of 68.60 years (range, 60-83 years). All the injured arms were dominant. 22 patients suffered from medial comminution. The mean angle of the postoperative humeral neck-shaft was 130.15 degrees. According to the postoperative humeral neck-shaft angle, there were 45 patients with anatomical reduction, 1 patient with acceptable reduction, and 1 patient with malreduciton. More demographic characteristics data of the two group were listed Table 1. There was no statistical significance between the two groups in the demographic characteristics (P>0.05, Table 1).

The average follow-up was 35.2 months (range, 24-48 months) in Group I and 33.5 months (range, 24-48 months) in Group II. The rates of varus malunion, screw perforation, and loss of reduction>5mm were significantly higher in Group I than in Group II (P<0.05). There was no statistical significance between the two groups in the rates of avascular necrosis (P>0.05). In general, the group with LCP and fibular allograft had 2 varus malunion and 1 screw perforation in comparison to a large number in the LCP alone group. The rate of total complications was significantly higher in Group I than in Group II (P<0.05). More details were listed in Table 2.

**Figure 3:**
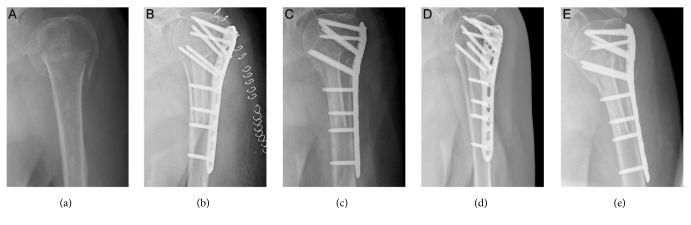
Radiograph of an 80-year-old woman with 4-part PHF treated by locking compression plate with intramedullary fibular allograft. (a) Preoperative X-ray film; (b) postoperative X-ray film; (c) X-ray film 3 months after operation; (d) X-ray film 6 months after operation; (e) X-ray film 18 months after operation.

**Figure 4:**
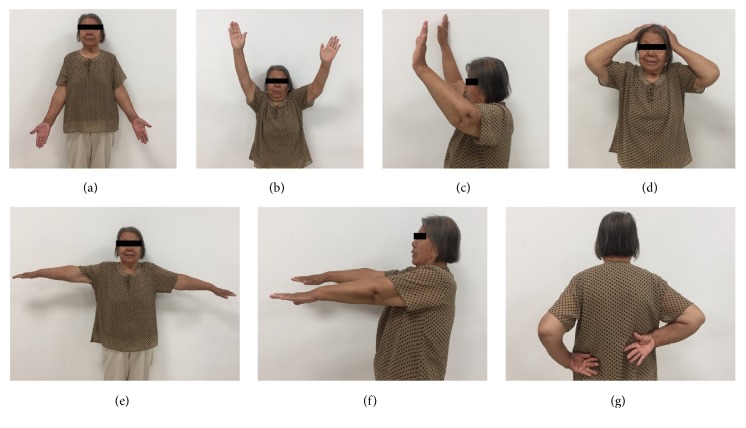
Function recovery of the patient in Figure 3 at follow-up period.

DASH in patients of Group I in was significantly higher than patients in Group II and CMS scores of patients of Group I were significantly lower than those of patients in Group II (P<0.05). Furthermore, our clinical results rated by the patients' subjective evaluation showed that excellent and good function in Group II was significantly higher than that in Group I(P<0.05) (Figures 3 and 4). More details were listed in Tables 2 and 3.

## 4. Discussion

**Figure 5:**
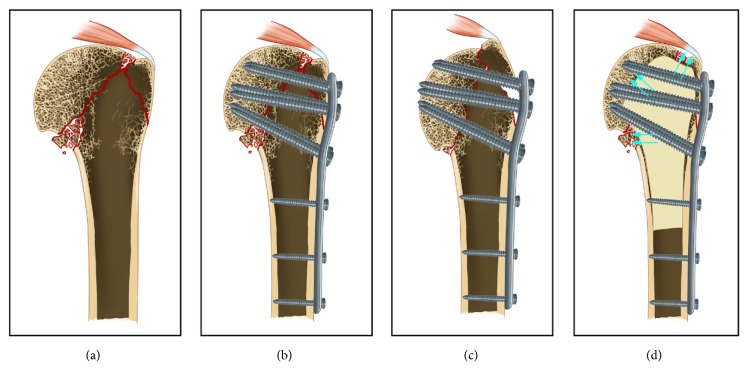
The schematic diagram of the major effect of fibular allograft (the fracture line marked by red color). (a) The picture presented a coronal plane of elderly osteoporotic PHF. The bone volume in humeral head was nearly empty; (b) the PHF was treated by LCP alone; (c) the retroversion of humeral head, medial, and upward shift of the greater tuberosity, and the humeral head varus occurred due to lack of enough support effect; (d) the PHF was treated by LCP with fibular allograft, and the fibular allograft could provide enough support effect, and the arrows showed the support effect for humeral head, greater tuberosity, and medial cortical bone.

This is a retrospective comparative clinical study of LCP alone and LCP with fibular allograft in the treatment of elderly displaced PHF. The present study showed that patients treated by LCP with fibular allograft had a better functional outcome and a lower complication rate in comparison to patients treated by LCP alone. Therefore, fibular allograft played an important role in the treatment of elderly displaced PHF, especially in the patients with osteoporosis. In our clinical experience, there are four major effects of fibular allograft as follows (Figure 5): (1) fibular allograft as volumetric filling in the bone void could prevent the humeral head to retreat after LCP fixation; (2) fibular allograft could provide enough medial stability and thus avoid the humeral head varus; (3) fibular allograft, especially in anatomical type, could provide the support for greater tuberosity, which is good for the restoration of the shape of greater tuberosity so as to improve the abduction function of shoulder; (4) fibular allograft would not disturb the blood supply of the humeral head, and, on the contrary, it provides a stable surface that could allow osteogenic tissue across the fracture site and then accelerate the fracture healing.

Although the optimal surgical management of elderly displaced PHF has not been determined, most of surgeons believed that LCP is a promising treatment for PHF. There are several advantages for LCP system of the proximal humerus, such as anatomic design, divergent angulated configuration of locking screws, and high rotational and angular stability [48]. However, a high complication rate has been reported in the treatment of PHF by using LCP alone [9]. Therefore, researchers began to realize the importance of medial support and BMD [11, 30, 48–50]. They found that lack of medial support and a low BMD were associated with a higher risk of loss of reduction and a poor clinical result after LCP fixation. Gardner et al. were the first to describe the importance of medial support in the treatment of PHF by LCP [30]. They conducted that medial support screws played a key role in LCP fixation of displaced PHF, but a biomechanical study showed the addition of medial support screws had no effect on the stiffness of the medial cortex in cases with varus malunion [51]. In addition, a clinical study demonstrated that the place of calcar screws may be cause a high risk of humeral head necrosis [11]. Some investigators tried to use bone cement to strengthen the stability of LCP system in PHF, and the clinical data showed that a good clinical result with a decreasing complication rate [31], but the cement-related heat injuries may exist [34]. The technique of additional medial plate or bone autograft was also attempted to solve the problem of the medial stability, and some drawbacks were existed, such as demanding technique, neurovascular injuries and donor-site morbidity [32, 33, 36]. Our previous study showed that elderly patients with displaced PHF could obtain a satisfied clinical outcome by LCP with fibular allograft [40], the other two clinical studies also presented a similar clinical result as our precious study [52, 53]. In order to investigate the effect of fibular allograft in elderly patients with displaced PHF, we compared the two methods of LCP alone and LCP with fibular allograft, and a better clinical result with a lower complication rate were found in patients treated by LCP with fibular allograft, which was also consistent with the biomechanical data [41–44].

A previous study showed that the rate of screw cut-out was up to 43% in patients order than 60 years [10], so they believed that the bone quality and quantity of the humeral head played a key role in obtaining stable fixation. Xavier et al. thought that cavity defects in proximal humerus were an indication to abandon internal fixation and to choose the method of prosthetic replacement [54]. So recently, hemiarthroplasty has been recommended as a good option for the treatment displaced PHF, especially with elderly patients [55–57]. However, high complication rates and poor functional results have been reported by the method because of tuberosity malreduction or migration, postoperative pain and instability [58, 59]. In our study, we found that patients treated by LCP alone also had a high complication rate of screw perforation and varus malunion, even if calcar screws were added. We also detected that some patients treated by LCP alone had a problem of shoulder abduction weakness due to the greater tuberosity absorption or migration. The aforementioned problems rarely existed in patients through LCP with fibular allograft in our study. We thought that void filler played a critical role in decreasing the complication rates of screw perforation and varus malunion, and improving the shoulder abduction function, because suitable void filler could provide a satisfied mechanical environment for the head fragment, medial cortical bone and greater tuberosity.

To the best of our knowledge, this is the first comparative study about LCP alone and LCP with fibular allograft in the treatment of elderly displaced PHF. Large amounts of information on the characteristics of patients, treatment outcomes, complications and clinical experience were described in our study. However, there were some limitations in the current study. The number of patients was relatively small and our study was retrospective in nature. Moreover, the operations were performed by three orthopedic experts who maybe have a preference in treatment options, and all the data of patients were collected from a single-center. Therefore, more multi-center prospective randomized controlled trails are needed to overcome these limitations.

In conclusion, the present results showed that that patients treated by LCP with fibular allograft had a better functional outcome and a lower complication rate comparted to patients treated by LCP alone. Suitable void filler in the proximal humerus for supporting the head fragment, medial cortical bone and greater tuberosity might play a key role in reducing the incidence of the complications in elderly patients, especially with osteoporosis.

## Figures and Tables

**Table 1:** Demographic characteristics data for patients included in this study.

Characteristic	Group I (n=42)	Group II (n=47)	P-value
Average age (year)	69.12	68.60	0.524
Sex distribution (male : female)	15 : 27	12 : 35	0.359
Dominant arm involvement	14 : 28	18 : 29	0.664
The mechanism of injury (F: TA)	37 : 5	40 : 7	0.680
Dual mineral absorptiometry	-3.0	-2.52	0.335
Classification of Neer (3 part : 4 part)	10 : 32	12 : 35	0.851
Medial comminution	18	22	0.708
The mean time from injury to surgery (day)	6.60	6.74	0.585
Intraoperative bleeding volume (ml)	219.52	232.77	0.332
Neck-shaft angle (degree)	128.71	130.15	0.450
Anatomical reduction	34	45	0.056
Acceptable reduction	7	1	
Malreduction	1	1	

F: fall, TA: traffic accident.

**Table 2:** Functional outcomes and complications of patients in two groups.

Variable	Group I (n=42)	Group II (n=47)	P-value
>5mm loss of reduction	9	2	0.022
Functional outcomes			
DASH	49.00	36.17	0.001
CMS	70.29	74.38	0.020
Complications			
Varus malunion	8	2	0.042
Screw perforation	6	1	0.049
Avascular necrosis	3	5	0.717
Total complications	17	8	0.018

**Table 3:** Patients' subjective evaluation of clinical results at 24 months.

Groups	Excellent	Good	Fair	Poor	P-value
Group I	23	15	3	1	0.001
Group II	43	4	-	-	

## Data Availability

Our data used to support the findings of this study are included within the article.
